# Frequency and intensity of [^18^F]-PSMA-1007 uptake after COVID-19 vaccination in clinical PET

**DOI:** 10.1259/bjro.20210084

**Published:** 2022-09-09

**Authors:** Alexander Maurer, Helen Schiesser, Stephan Skawran, Antonio G. Gennari, Manuel Dittli, Irene A. Burger, Cäcilia Mader, Christoph Berger, Daniel Eberli, Martin W. Huellner, Michael Messerli

**Affiliations:** ^1^ Department of Nuclear Medicine, University Hospital Zurich, Zurich, Switzerland; ^2^ University of Zurich, Zurich, Switzerland; ^3^ Department of Nuclear Medicine, Kantonsspital Baden, Baden, Switzerland; ^4^ Division of Infectious Diseases and Children’s Research Centre, University Children’s Hospital Zurich, Zurich, Switzerland; ^5^ Department of Urology, University Hospital Zurich, Zurich, Switzerland

## Abstract

**Objectives:**

To assess the frequency and intensity of [^18^F]-prostate-specific membrane antigen (PSMA)-1007 axillary uptake in lymph nodes ipsilateral to COVID-19 vaccination with BNT162b2 (Pfizer-BioNTech) or mRNA-1273 (Moderna) in patients with prostate cancer referred for oncological [^18^F]-PSMA positron emission tomography (PET)/CT or PET/MR imaging.

**Methods:**

126 patients undergoing [^18^F]-PSMA PET/CT or PET/MR imaging were retrospectively included. [^18^F]-PSMA activity (maximum standardized uptake value) of ipsilateral axillary lymph nodes was measured and compared with the non-vaccinated contralateral side and with a non-vaccinated negative control group. [^18^F]-PSMA active lymph node metastases were measured to serve as quantitative reference.

**Results:**

There was a significant difference in maximum standardized uptake value in ipsilateral and compared to contralateral axillary lymph nodes in the vaccination group (*n* = 63, *p* < 0.001) and no such difference in the non-vaccinated control group (*n = 63, p* = 0.379). Vaccinated patients showed mildly increased axillary lymph node [^18^F]-PSMA uptake as compared to non-vaccinated patients (*p* = 0.03). [^18^F]-PSMA activity of of lymph node metastases was significantly higher (*p* < 0.001) compared to axillary lymph nodes of vaccinated patients.

**Conclusion:**

Our data suggest mildly increased [^18^F]-PSMA uptake after COVID-19 vaccination in ipsilateral axillary lymph nodes. However, given the significantly higher [^18^F]-PSMA uptake of prostatic lymph node metastases compared to “reactive” nodes after COVID-19 vaccination, no therapeutic and diagnostic dilemma is to be expected.

**Advances in knowledge:**

No specific preparations or precautions (*e.g.* adaption of vaccination scheduling) need to be undertaken in patients undergoing [^18^F]-PSMA PET imaging after COVID-19 vaccination.

## Introduction

The COVID-19 pandemic still deeply affects healthcare systems worldwide. The harmful effect the virus exerts is a major concern for global health, particularly in elderly patients or patients with malignant tumors.^1^ Among the latter, the fatality rate was reported to be higher even after adjusting for confounders.^1^ This is why patients with malignant diseases were prioritized to receive COVID-19 vaccinations. Swelling and pain at the injection site as well as ipsilateral axillary lymphadenopathy were acknowledged as side-effects of COVID-19 vaccines.^2^

Numerous studies reported metabolically active axillary lymph nodes after [^18^F]-fludeoxyglucose (FDG) positron emission tomography/computed tomography (PET/CT),^3,4^ sometimes with relevant clinical impacts such as unnecessary lymph node core needle biopsy.^5^ Therefore, different expert consensus statements for the management of axillary lymph adenopathy in patients with prior COVID-19 vaccination undergoing imaging were published.^6^ However, previous studies were mainly focused on [^18^F]-FDG-imaging^3^ and evidence is sparse with regard to the potential effect of COVID-19 vaccination in prostate-specific membrane antigen [^18^F]-PSMA-1007 imaging.

Accordingly, the purpose of this study was to analyze the overall frequency and intensity of [^18^F]-PSMA avid axillary lymph nodes ipsilateral to COVID-19 vaccine injection site in patients with prostate cancer.

## Methods and material

### Study design and patients

**Figure 1.**
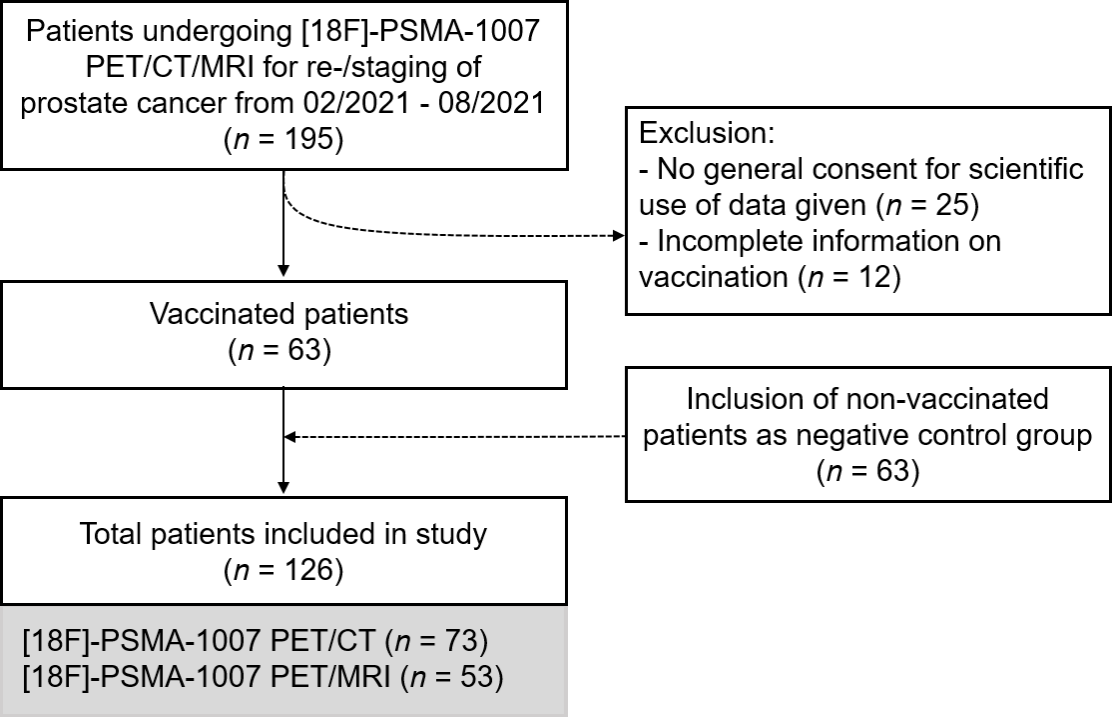
Study flowchart. PET/CT, positron emission tomography/CT; PSMA, prostate-specific membrane antigen.

In this retrospective single-center study, we included all patients who underwent clinically indicated [^18^F]-PSMA-1007 PET/CT or PET/MRI for staging or restaging of prostate cancer during the study period from February 2021 to August 2021. Thereof, 63 patients had their first or second vaccination with either BNT162b2 (Comirnaty^®^, Pfizer/BioNTech, New York, USA/Mainz, Germany) or mRNA-1273 (Moderna^®^, Moderna Biotech, Cambridge, USA) before the PET scan. Dates of the first and, if applicable, second vaccination as well as the vaccine administered were recorded. An additional 63 non-vaccinated patients were included consecutively and served as negative control group. A flow chart of the study is presented in Figure 1. The result was a total of 126 patients. Patient characteristics and clinical information including age, body mass index (BMI), initial tumor stage (TNM classification), prostate-specific membrane antigen (PSA) value less than 4 weeks before scan and indication for imaging were recorded.

Written informed consent for the scientific use of medical data was obtained from all patients. The study was approved by the local ethics committee (Trial No. 2021–00444) and was conducted in compliance with ICH-GCP rules and the Declaration of Helsinki.

### PET acquisition and image reconstruction

Examinations were performed on a latest generation PET/CT scanner (GE Discovery MI, GE Healthcare, Waukesha, WI) or PET/MR scanner (SIGNA, GE Healthcare) using a standardized clinical [^18^F]-PSMA dosage protocol as described in detail.^7^ The injected dose of [^18^F]-PSMA was 3 MBq/kg (mean ± SD, 242 ± 35 MBq; range 130–349 MBq), uptake time was 90 min. PET reconstructions were generated using penalized likelihood reconstruction (Q.Clear, GE Healthcare) with a *β*-value of 450. All PET data sets were reconstructed with a 256 × 256 pixel matrix.

### PET data analysis

One reader (A.M., 6 years of experience in radiology, and 3 years of experience in PET imaging) reviewed all PET data sets. Commercial image analysis software (Advantage Workstation v. 4.7, GE Healthcare) was used for review. The reader measured [^18^F]-PSMA avidity by drawing a semi-automated cubicle volume of interest (VOI) around the most avid axillary lymph nodes bilaterally. [^18^F]-PSMA avidity was measured as the maximum standardized uptake value (SUV_max_) within the VOI (*i.e.* decay corrected radioactivity per volume [kBq / ml], divided by the initially injected dose [MBq] and multiplied by body weight [kg]). The absolute difference in SUV_max_ between the data pairs of axillary lymph nodes was calculated for the vaccinated group. A positive reaction was defined as unilateral [^18^F]-PSMA avidity of axillary lymph nodes ipsilateral to the prior vaccination site having a difference in SUV_max_ of >1.0 (avidity ipsilateral lymph nodes - avidity contralateral lymph node), as previously described.^5^

SUV_max_ of ipsilateral and contralateral axillary lymph nodes in the vaccinated group and SUV_max_ of right and left axillary lymph nodes in the non-vaccinated group were compared. Further, we reviewed all clinical reports, and SUV_max_ of up to three [^18^F]-PSMA avid lymph node metastases were measured to serve as quantitative reference. SUV_max_ of left axillary lymph nodes in the non-vaccinated group served as a negative control.

### Statistical analysis

All statistical analyses were performed in the open-source statistics software R (v. 3.6.1, R Foundation for Statistical Computing, Vienna, Austria). Categorical variables are expressed as frequency distribution. Continuous variables are presented as mean ± standard deviation if normally distributed, or median (range) otherwise. Assessment of group differences was determined using *t*-test (paired or unpaired) after ensuring a normal distribution of the data using the Shapiro–Wilk test, and normality was rejected if *p* < 0.05. For non-normally distributed data, Wilcoxon-test or Mann–Whitney test was used. Categorical data were compared using χ^2^ test. For all comparisons, a two-tailed *p*-value of <0.05 was considered to be statistically significant.

## Results

### Patient characteristics and clinical information

126 patients undergoing [^18^F]-PSMA-1007 PET/CT (73/126, 58%) or PET/MR imaging (53/126, 42%) for staging or restaging of prostate cancer were retrospectively included in the study, see Table 1. 29 of the 63 vaccinated patients (44%) had received Pfizer-BioNTech and 34 (56%) Moderna vaccines. 11 of 63 patients (17%) were vaccinated for the first time, and 52 of 63 patients (83%) had been vaccinated two times.

**Table 1.** Demographic data of study subjects (*n* = 126)

Age, years	71 ± 8 (53–87)
Body weight, kg	82 ± 12 (54 - 115)
Body height, m	1.76 ± 0.07 (1.52–1.89)
BMI, kg/m^2^	26.6 ± 3.6 (19.2–34.9)
PSA values, ng/ml	2.9 (IQR 0.59–9.91)
Indication for imaging	
Staging	36 (29%)
Early BCR	37 (30%)
Tumor burden evaluation	52 (41%)
Initial T classification	
T1	21 (22%)
T2	34 (35%)
T3	39 (41%)
T4	2 (2%)
Initial N classification	
N0	110 (87%)
N1	16 (13%)
Initial M classification	
M0	115 (91%)
M1	11 (9%)

IQR interquartile range; BCR, biochemical recurrence; BMI, Body mass index; MBq, Mega-Becquerel; PET/CT, positron emission tomography/computed tomography; PET/MRI, positron emission tomography/magnet resonance imaging.

Values are given as absolute numbers and percentages in parenthesis or mean ± standard deviation (range) or median (interquartile range).

## [^18^F]-PSMA avidity of axillary lymph nodes ipsilateral to COVID-19 vaccination site

**Figure 2.**
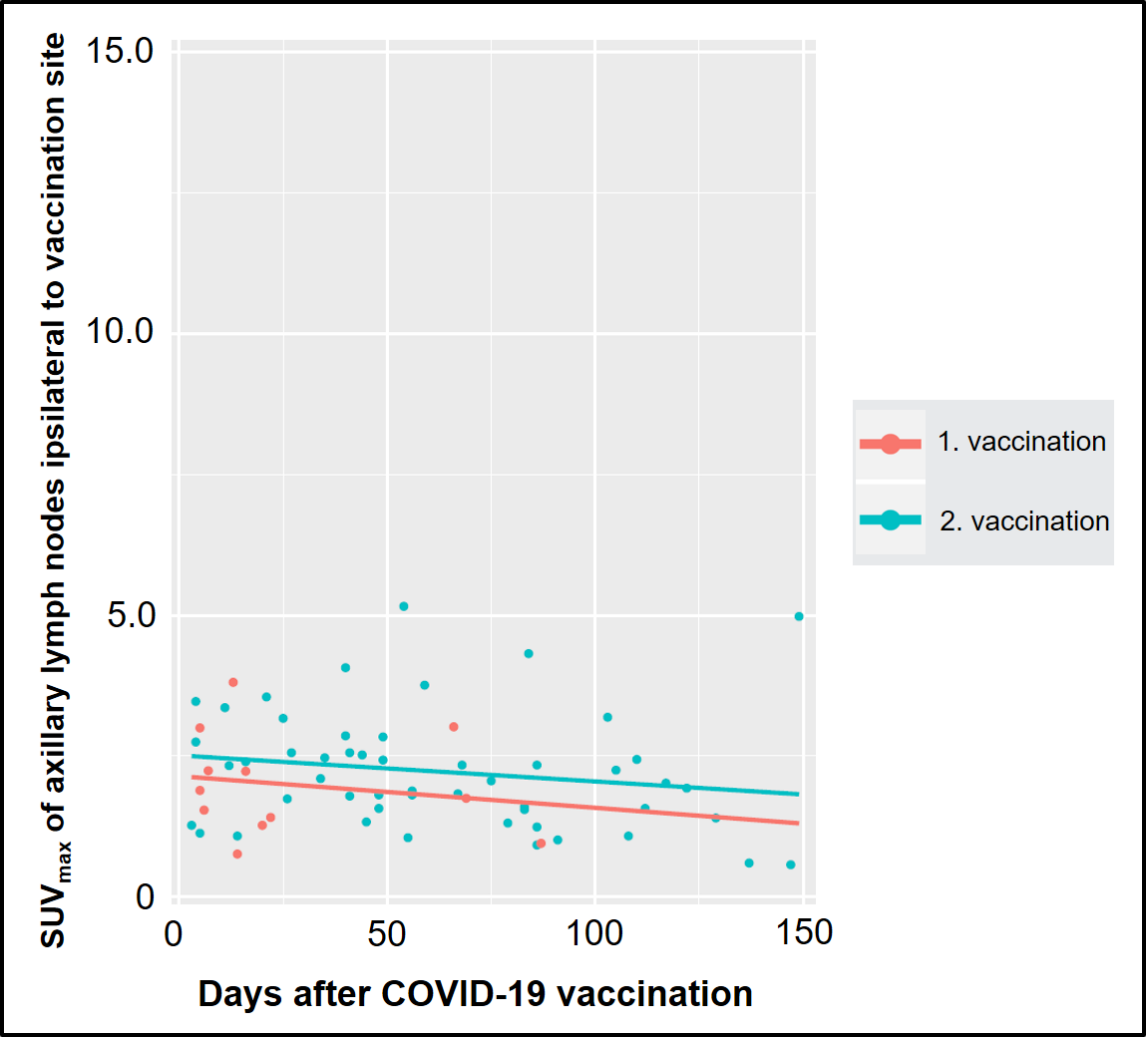
Scatter plot of SUV_max_ in vaccinated study patients (*n* = 63) undergoing [^18^F]-PSMA-1007 PET measuring lymph nodes ipsilateral to COVID-19 vaccination and separated for post first vaccination (red) and post second vaccination (green). PET, positron emission tomography; PSMA, prostate-specific membrance antigen; SUV_max_, maximum standardized uptake value.

Overall, there were 12/63 (19%) patients presenting with [^18^F]-PSMA avid lymph nodes ipsilateral to the vaccine injection site (SUV_max_ range, 1.9–6.9). Frequency of patients with [^18^F]-PSMA active ipsilateral lymph nodes after COVID-19 vaccination with regard to time after vaccination are presented in Table 2. Pearson correlation showed a negative, albeit not significant correlation of day after COVID-19 vaccination and SUV_max_ of ipsilateral lymph nodes (first vaccination: *R* = −0.18, *p* = 0.580, second vaccination: *R* = −0.18, *p* = 0.217, overall: *R* = −0.13, *p* = 0.297), Figure 2.

**Table 2.** Frequency of patients with [^18^F]-PSMA-1007-active^a^ ipsilateral lymph nodes after COVID-19 vaccination with regard to time delay between vaccination and PET scan

		Increaseda [^18^F]-PSMA-1007 uptake in axillary lymph node		
	**Total patients**	**Yes**	**No**	**SUV_max_**
Overall	63 (100%)	12 (17%)	51 (83%)	3.5 (1.9–6.5)
Day 0–14	13 (21%)	2 (15%)	11 (85%)	3.1 (2.3–3.8)
Day 15–28	8 (13%)	1 (12%)	7 (88%)	3.2 (3.2)
Day 29–50	12 (19%)	3 (25%)	9 (75%)	3.1 (2.4–4.1)
Day 51–100	19 (30%)	4 (21%)	15 (79%)	4.0 (1.9–6.5)
Day >100	11 (17%)	2 (18%)	9 (82%)	3.7 (2.4–4.9)

PET, positron emission tomography; SUVmax, Maximum standardized uptake value.

Values are given as absolute numbers and percentages in parenthesis or mean (range).

a *i.e.* defined as unilateral PSMA-activity of axillary lymph nodes ipsilateral to prior vaccination having a difference in SUV_max_>1.0 (avidity ipsilateral lymph nodes - avidity contralateral lymph node).

## Quantitative comparison of non-vaccinated vs. vaccinated patients and [^18^F]-PSMA avid lymph node metastases

There was a significant difference in SUV_max_ in ipsilateral and contralateral axillary lymph nodes in the vaccinated group, *p* < 0.001. No difference in SUV_max_ in left and right axillary lymph node in the non-vaccinated control group was detected, *p* = 0.379.

**Figure 3.**
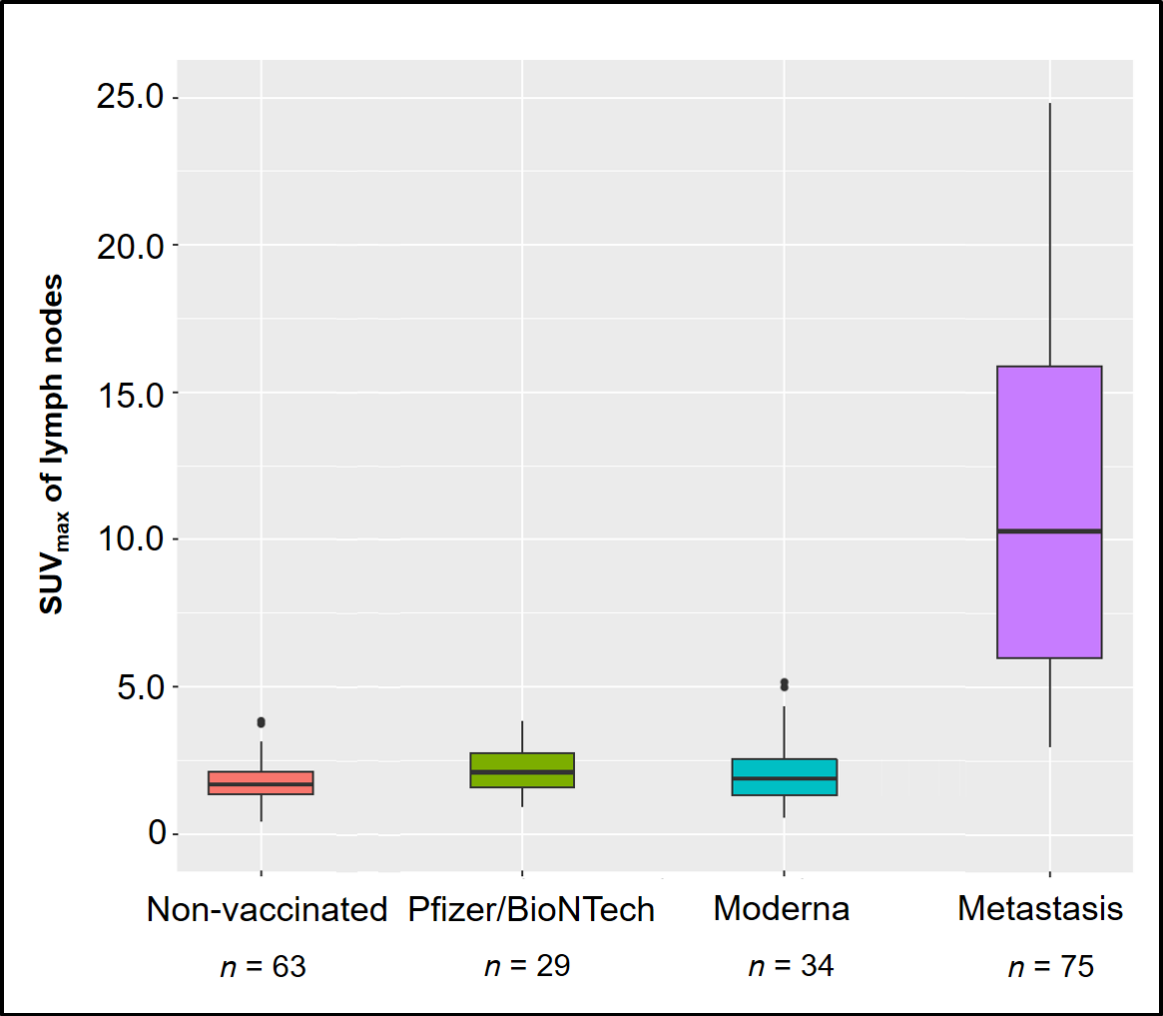
Box plots of [^18^F]-PSMA SUV_max_ of axillary lymph nodes in the negative control group, vaccinated group (*i.e.* lymph nodes ipsilateral to COVID-19 vaccination site) and lymph node metastasis of prostatic cancer. PSMA, prostate-specific membrance antigen; SUV_max_, maximum standardized uptake value.

The mean [^18^F]-PSMA uptake in left axillary lymph nodes of non-vaccinated patients was SUV_max_ 1.8 ± 0.7 (range, 0.4 to 3.8). Vaccinated patients showed mildly increased [^18^F]-PSMA uptake in the ipsilateral lymph nodes compared to non-vaccinated patients, with a mean SUV_max_ of 2.2 ± 1.0 (range, 0.6–5.2); *p* = 0.03. Comparing activity of [^18^F]-PSMA avid lymph node metastases (*n* = 75) with axillary lymph nodes of vaccinated patients, we observed a significantly higher [^18^F]-PSMA uptake of lymph node metastases, with a median SUV_max_ of 13.3 (range, 6.8–21.3), *p* < 0.001, Figure 3 **.**

**Figure 4.**
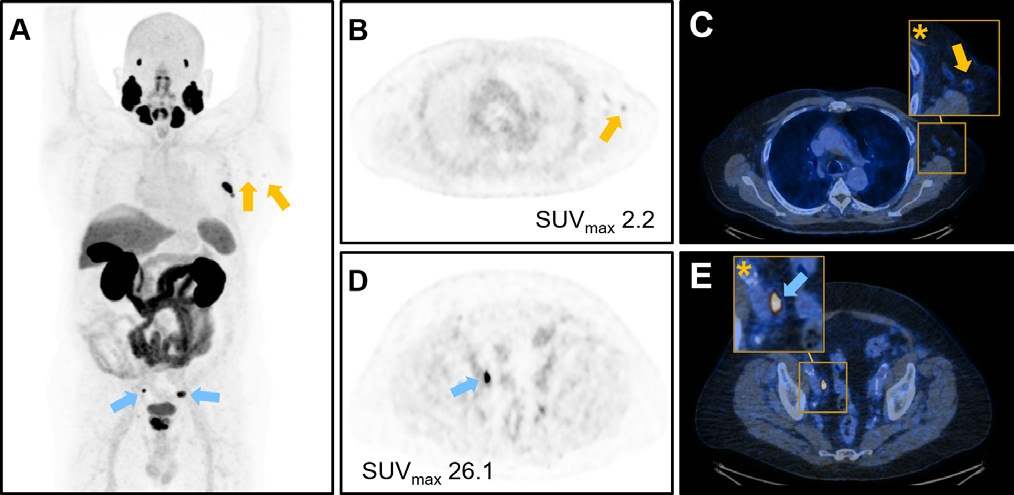
Representative images of a 69-year-old patient who underwent [^18^F]-PSMA-1007 PET/CT for staging of newly diagnosed prostate cancer with mildly [^18^F]-PSMA avid axillary lymphadenopathy (orange arrows) after second COVID-19 vaccination on the left side-with Pfizer-BioNTech. (**A**) Maximum intensity projection image showing [^18^F]-PSMA avid left axillary lymph nodes (orange arrows) as well as [^18^F]-PSMA avid pelvic lymph node metastases (blue arrows), (**B**) axial PET image of the axilla and (**C**) corresponding fused PET/CT images with magnified image of the left axilla (asterisk), (**D**) axial PET image of lymphe node metastases in the pelvic region and (**E**) corresponding fused PET/CT images with magnified image of the left pelvis (asterisk). PET, positron emission tomography; PSMA, prostate-specific membrance antigen; SUV_max_, maximum standardized uptake value.

A representative case of PET/CT with [^18^F]-PSMA avid axillary lymphadenopathy is given in Figure 4.

## Discussion

In this retrospective cohort study, we sought to assess frequency and intensity of axillary lymph node [^18^F]-PSMA-1007 uptake ipsilateral to COVID-19 vaccination in patients with prostate cancer undergoing PET.

The main findings of the study were as follows: first, there was mildly increased [^18^F]-PSMA uptake in ipsilateral lymph nodes after COVID-19 vaccination in comparison to the non-vaccinated control cohort. Second, [^18^F]-PSMA positive prostatic lymph node metastases had significantly higher [^18^F]-PSMA uptake than axillary lymph nodes after COVID-19 vaccination.

In contrast to our study, the recent work published by Eifer et al did not show a difference in [^18^F]-PSMA uptake in ipsilateral lymph nodes after COVID-19 vaccination.^8^ One possible explanation for this discrepancy may be that Eifer et al used two different radiotracers [^18^F]-PSMA and [^68^Ga]-PSMA in their study. To that regard, Rauscher et al demonstrated higher unspecific uptake in lymph nodes in [^18^F]-PSMA-1007 PET, the tracer used in our study, compared to [^68^Ga]-PSMA-11 PET.^9^ Further, a different study design using an SUV_max_ cut-off ratio of ≥1.5 in ipsilateral *vs* contralateral lymph nodes for defining positive lymph nodes lowers the frequency of “positive findings”.^8^

The mechanism of [^18^F]-PSMA expression and/or [^18^F]-PSMA ligand accumulation in inflammatory and infectious processes is not well understood, and only a few studies on this topic are available.^10^ There is some evidence that increased [^18^F]-PSMA expression during neovascularization,^11^ accumulation of [^18^F]-PSMA ligands at the infection site^10^ and the interference of macrophage folate receptors with [^18^F]-PSMA ligands^12^ may play a role in inflammatory and infectious processes.

Pelvic and retroperitoneal lymph nodes are the most common site for nodal prostate cancer metastases.^13^ In advanced disease, however, thoracic and cervical lymph nodes may be involved as well.^13^ Axillary lymph node metastases from prostate cancer are extremely rare, yet a few cases have been described in literature.^13–15^ Therefore, increased [^18^F]-PSMA uptake in axillary lymph nodes after COVID-19 vaccination bears little impact in clinical routine, especially as lymph node metastases of prostate carcinoma show significantly higher [^18^F]-PSMA ligand accumulation than axillary lymph nodes after COVID-19 vaccination, as observed in our study. In contrast, Skawran et al demonstrated metabolically active axillary lymph nodes in [^18^F]-FDG PET after COVID-19 vaccination leading to diagnostic and therapeutic dilemmas.^5^

We are aware of some noteworthy limitations: first, our study population is rather small. Second, no pathological confirmation of the inflammatory state of axillary lymph nodes after COVID-19 vaccination was available, further yielding the potential bias of additional different pathologies and/or haematological disorder. Third, there is no follow-up imaging to assess the persistence of [^18^F]-PSMA uptake. Fourth, because of the relatively small study population, no subanalysis for the two used vaccines BNT162b2 and mRNA-1273 and for the different scanner types (PET/CT or PET/MR) was performed. Fourth, we did not include patients after third (*i.e.* booster) vaccinations, which may also differently impact lymph node activity. Fifth, we did not measure additional reference tissue and/or different sites of reactive PSMA-uptake which may serve in future studies as an additional quantitative comparison.

## Conclusions

Our data suggest (mildly) increased [^18^F]-PSMA uptake after COVID-19 vaccination in ipsilateral axillary lymph nodes - possibly owing to reactive inflammatory changes. Given the significantly higher [^18^F]-PSMA uptake of prostatic cancer lymph node metastases compared to “reactive” nodes after COVID-19 vaccination and the overall sparsity of axillary prostate cancer lymph node metastases, no therapeutic and diagnostic dilemma is to be expected.
